# A Simple and Practical Solvent-Free Preparation of Polymaleimide 

**DOI:** 10.3390/molecules16031981

**Published:** 2011-02-28

**Authors:** Xing-Rong Zhang, Bing-Tao Tang, Shu-Fen Zhang

**Affiliations:** State Key Laboratory of Fine Chemicals, Dalian University of Technology, Dalian 116024, China

**Keywords:** polymaleimide, polymaleic anhydride, urea, solvent-free reaction

## Abstract

Polymaleimide (PMAI) was synthesized by reacting polymaleic anhydride (PMA) with urea via a solvent-free reaction at 180 °C. The conversion of PMA could reach 95%. This method is simple, practical and environmentally-friendly. The structure of the resulting PMAI was characterized by ^1^H-NMR and IR.

## 1. Introduction

Polymaleimide (PMAI) is a type of polymer with high reactivity, often used as the polymeric skeleton of many functional materials with excellent thermal stability. For example, PMAI was used by Ganjadhara *et al.* to prepare nonlinear optical materials as liquid crystals to improve their thermal properties [1]. Generally, PMAI is synthesized by two approaches: one is homopolymerization of maleimide by free radical or anionic polymerization [2,3]. The other is thermolysis [4] or hydrolysis [5] of substituted maleimide homopolymers. In both methods, maleimide is used as the starting material, but as we know, maleimide is expensive, relatively unavailable and difficult to purify. Moreover, in the second method, the additional reaction steps make the synthesis more difficult. 

Herein, we report a simple and practical synthetic approach to PMAI using maleic anhydride MA), which is much cheaper than maleimide, as raw material. In this approach, polymerization of maleic anhydride under mild conditions is carried out first to prepare polymaleic anhydride (PMA) and the latter then reacts with an imidization agent to produce PMAI. The selection of a suitable imidization agent and reactions conditions is essential for the successful synthesis of PMAI. Conventional applicable imidization agents include ammonium formate, hydroxylamine hydrochloride, ammonia gas or ammonia and urea [6]. All these agents release ammonia at high temperature, and then this ammonia further reacts with the anhydride to form an imide. In these imidization reactions, organic solvents such as DMF or xylene which are difficult to remove after the reaction are typically used. In addition, when ammonium formate or hydroxylamine hydrochloride were used as imidization agents, formic acid and hydrogen chloride which are harmful to the environment were produced; when ammonia gas or ammonia were used as an imidization agent, rigorous conditions, including high temperatures and pressures were required. On the other hand, when urea was used the by-products were H_2_O and CO_2_, which are not harmful to the environment. Consequently, in this study, urea was chosen as imidization agent, and a solvent-free reaction procedure was designed to realize a synthesis of PMAI that is much more convenient, economical and environmentally friendly.

## 2. Results and Discussion

**Scheme 1:**
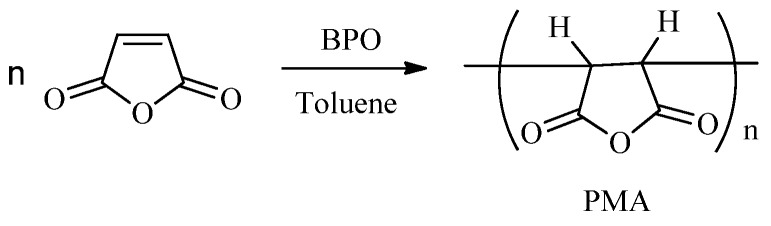
Synthesis of polymaleic anhydride (PMA).

PMA was synthesized in anhydrous toluene in a reaction initiated by benzoyl peroxide (BPO) [7] (Scheme 1). The weight-average molecular weight (M*w* = 1.0 × 10^3^) of the PMA was obtained using an Agilent 1200 series gel permeation chromatography (GPC) (eluent: THF, flow rate: 1 mL/min). 

**Scheme 2:**
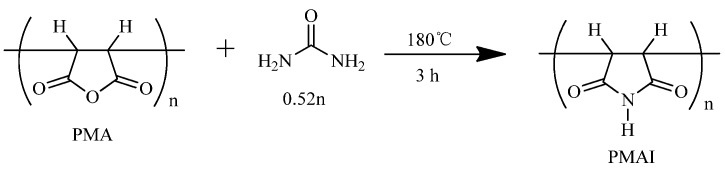
Synthesis of polymaleimide (PMAI).

Maleic anhydride content (in mass) in PMA was 80.3% calculated from the PMA elemental analysis result: (C: 56.72, H: 3.748, O: 39.34) according to formula (1). PMAI was then prepared from PMA (Scheme 2) via a solvent-free reaction.

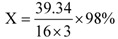
(1)

*Notes:* X: maleic anhydride content (in mass) in PMA; 39.34: oxygen content (in mass) in PMA; 16: the molar mass of oxygen; 3: the number of oxygen in MA; 98: the molar mass of MA.

**Figure 1:**
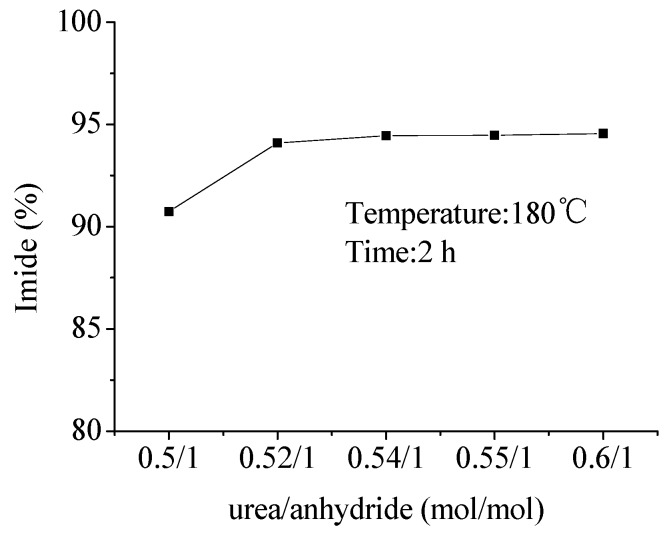
Effects of the amount of urea on the content of imide.

**Figure 2:**
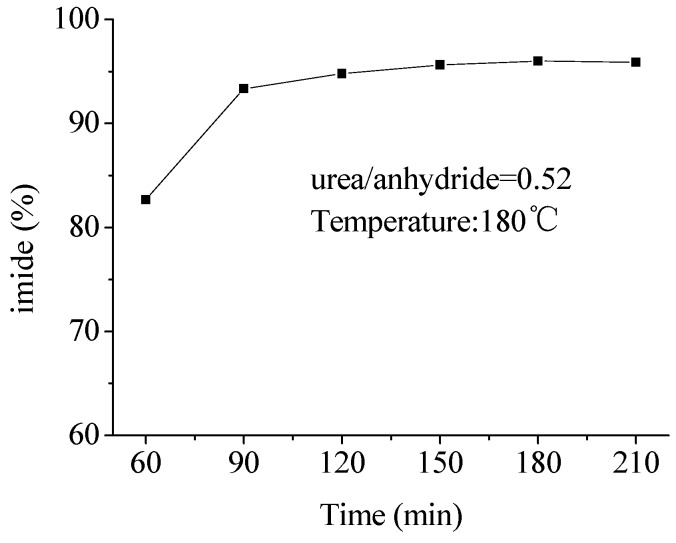
Effects of the reaction time on the content of imide.

PMAI was then prepared from PMA via a solvent-free reaction (Scheme 2). Factors affecting the solvent-free reaction (Scheme 1) of PMA and urea were temperature, amounts of urea and reaction time. The effects of those factors on imide content were shown in Table 1, Figure 1 and Figure 2, respectively.

Generally, an amide or imide would be obtained when anhydride react with imidizing agents. According to the literature [8], imide was formed at a temperature of 140 °C or higher. In view of the decomposition temperature of urea (160 °C) and the softening temperature of PMA (120 °C), temperatures higher than 160 °C were studied and the results are shown in Table 1. In this study, the mixture began to soften at 92 °C, which was observed by a micro melting point apparatus, and it softened completely at 110 °C. This softening state was beneficial to the reaction between PMA and urea. It was clear in Table 1 that the content of imide was constant when the temperature was higher than 180 °C. So the temperature of 180 °C was selected.

**Table 1:** Imide content of the products under different temperatures.

Temp (°C)	Content of imide (mmol/g)	Imide/anhydride (%)
160	6.72	81.4
170	7.69	93.1
180	7.89	95.5
190	7.90	95.6
200	7.90	95.6

*Notes:* 1. Conditions: urea/anhydride = 0.52, Time: 3 h; 2. Theoretical content of imide: 8.26 mmol/g.

The effect of the amount of urea on the content of imide was also investigated (Figure 1). Theoretically, 1 mole of urea can react with 2 moles of anhydride to yield 2 moles of imide. In consideration of the loss of urea in the solvent-free reaction, addition of more urea was studied. With the increase of the amount of urea, the content of imide increased. When the ratio of urea to anhydride was 0.52, the content of imide was 94%. When the ratio was further increased, the content of imide did not increase any more. This indicated that under solvent-free high-temperature conditions, a little excess urea could improve the content of imide up to 94%. 

In this solvent-free reaction, reaction time must be enough to complete the reaction, so reaction times of 1 h, 1.5 h, 2 h, 2.5 h, 3 h and 3.5 h were selected to investigate the effect on the content of imide. It is clear from Figure 2 that the content of imide reached 95% and was unchanged after 3 h when the reaction was complete, so it was not necessary to prolong the reaction time further.

**Figure 3:**
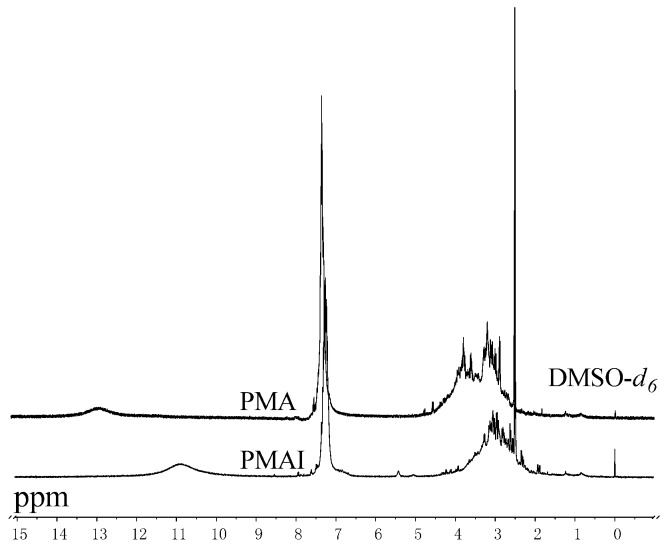
^1^H-NMR spectra of PMA and PMAI.

**Figure 4:**
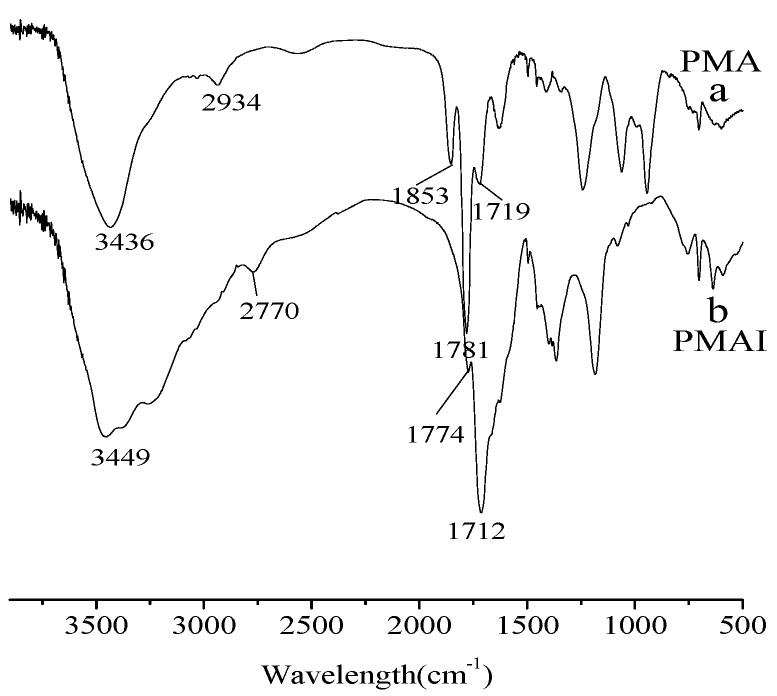
IR spectra of PMA and PMAI.

The product was characterized by ^1^H-NMR and IR as shown in Figure 3 and Figure 4, respectively. In Figure 3, the broad peak observed at 10.8 ppm was assigned to the imide proton [9]. The peaks between 6.7 and 7.6 ppm were attributed to the phenyl protons which were attached to the main PMA chain and produced by the BPO initiator when PMA was synthesized. There were no obvious peaks (12.8 ppm) due to carboxylic acid [9] in PMAI, compared to the starting material (PMA) because the ring-closure reaction occurred at 180 °C. 

Figure 4 gives the IR spectra of PMA (Figure 4a) and PMAI (Figure 4b). The peaks at 1,712 cm^−1^ and 1,774 cm^−1^ in Figure 4b are attributed to the C=O stretching vibration of succinimide, whichconfirmed the existence of PMAI. The peaks at 1,781 cm^−1^ and 1,853 cm^−1^ in Figure 4a assigned to C=O stretching vibration of succinic anhydride disappeared in Figure 4b. At the same time, there were no amide C=O stretching vibration peaks in Figure 4b. The peaks at 2,934 cm^−1^ in Figure 4a and 2,770 cm^−1^ in Figure 4b were assigned to the C-H stretching vibrations of succinic anhydride and succinimide in the main chain, respectively.

## 3. Experimental

### 3.1. General

Infrared spectra (IR) were recorded using a NEXUS EURO FT-IR spectrophotometer. ^1^H-NMR spectra were obtained with a Varian INOVA-400 using DMSO-*d*_6_ as solvent and TMS as the internal standard. Elemental analysis (EA) was performed on a Vario EL III Elemental Analyzer. An Agilent 1200 series gel permeation chromatography (GPC) was used for measuring the molecular weight of the polymaleic anhydride.

### 3.2. Synthesis of polymaleic anhydride (PMA)

The homopolymerization recation was carried out in a 500-mL, three-necked flask using maleic anhydride (50 g) and benzoyl peroxide (BPO, 5 g) in anhydrous toluene (200 mL) [7]. A representative feed was BPO/MA = 10/100 (in mass ratio). Usually, the reaction was stirred at 70 °C while the initiator was titrated into the reaction mixture over 30 min, and then elevated at 90–95 °C for 10 h. The polymer was precipitated in toluene at room temperature (20 °C), then, the upper toluene layer was removed. Anhydrous butanone (10 mL) was added into the polymer and stirred for 1 h at 90 °C, after that, the solution was quickly poured into anhydrous toluene (500 mL) at 60 °C. The PMA obtained was washed three times with anhydrous toluene, and then dried under vacuum at 60 °C. The polymers were obtained as light brown powders.

### 3.3. Solvent-free preparation of polymaleimide (PMAI)

PMAI was prepared from PMA and urea. PMA (1.00 g) and urea (0.26 g) were ground in a mortar. Then, the ground mixture was placed at an oven at 180 °C for 3 h. After cooling to room temperature, the reaction mixture was placed into ethanol overnight and then filtered. The filter cake was dried under vacuum.

## 4. Conclusions

In conclusion, polymaleimide was synthesized by the reaction of polymaleic anhydride with urea at 180 °C via a solvent-free reaction. The conversion of polymaleic anhydride could reach 95%. The structure of polymaleimide was characterized by ^1^H-NMR and IR. The proposed method provides a simple, practical and economical synthesis of polymaleimide.

## References

[1] Gangadhara, Noel C., Thomas M., Reyx D. (1998). Synthesis and characterization of polymaleimides containing4-cyanobiphenyl-based side groups for nonlinear optical applications. J. Polym. Sci. Part A Polym..

[2] Haas H.C., Moreau R.D. (1975). Maleimide polymers. III. Color reactions and kinetics. J. Polym. Sci., Polym. Chem. Ed..

[3] Agarwal P., Yu Q., Harant A., Berglund K.A. (2003). Synthesis and characterization of polymaleimide. Ind. Eng. Chem. Res..

[4] Otsu T., Matsumoto A., Tatsumi A. (1990). Synthesis and characterization of poly(*N*-tert-alkylmaleimide)s. Polym. Bull..

[5] Matsumoto A., Oki Y., Otsu T. (1992). Polymaleimides bearing a readily hydrolyzable side group: Synthesis and polymerization of *N*-trialkylsilylmaleimides and characterization of the polymers. Polym. J..

[6] Benjamin E., Hijji Y. (2008). The synthesis of unsubstituted cyclic imides using hydroxylamine under microwave irradiation. Molecules.

[7] Al-Roomi Y.M., Hussain K.F. (2006). Homo-oligomerization of maleic anhydride in nonpolar solvents: A kinetic study of deviations from nonlinear behavior. J. Appl. Polym. Sci..

[8] Vermeesch I.M., Groeninckx G., Coleman M.M. (1993). Poly(styrene-co-N-maleimide) copolymers: preparation by reactive extrusion, molecular characterization by FTIR, and use in blends. Macromolecules.

[9] Shi L., Chen S., Huang J. (2000). Radical copolymerization of α-(n-propyl)acrylic acid with maleimide in dioxane. Eur. Polym. J..

